# Autotitrator based on an Arduino Open Source Pump

**DOI:** 10.1016/j.ohx.2023.e00464

**Published:** 2023-08-07

**Authors:** Teresa del Castillo-Santaella, Julia Maldonado-Valderrama, Miguel Angel Fernandez-Rodriguez

**Affiliations:** a Department of Applied Physics, Faculty of Sciences, University of Granada, Campus de Fuentenueva s/n, 18071 Granada, Spain; b Department of Physical Chemistry, Faculty of Pharmacy, University of Granada, Campus de Cartuja s/n, 18071 Granada, Spain; c Excellence Research Unit “Modeling Nature” (MNat), University of Granada, Granada, Spain

**Keywords:** Arduino, Automated titration, pH meter

## Abstract

Acid–base titration is a quantitative analysis that enables knowing the quantity of acidic or basic groups present in a solution sample. It consists in the addition of base or acid to the solution sample while monitoring the pH to reach a neutral pH. The titration can be automated and here we present a low cost Arduino based Open Source Pump (OSPump) modified to act as an automated titrator with an obsolete but reliable Metrohm 713 pH meter. Our device is 50 times less expensive than second hand units from the pH meter manufacturer and inherently open to customization. We present two validation cases of study, including the lipolysis of a vegetable olive oil in water emulsion, characterized by the OSPump Titrator.

## Graphical abstract

**Figure:**
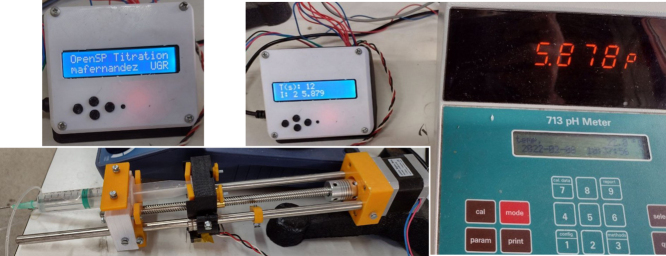

**Table:** 

Specifications table	

Hardware name	OSPump Titrator

Subject area	•*Biological sciences (e.g. microbiology and biochemistry)*
Hardware type	•*Measuring physical properties and in-lab sensors*
Closest commercial analog	Metrohm Titrando
Open source license	CC BY-SA 4.0
Cost of hardware	100 €
Source file repository	https://doi.org/10.5281/zenodo.7971517

## 1 Hardware in context

The necessity to judge the acidity of liquid solutions accompanies humanity since the beginning of civilization. It was necessary to judge whether a vinegar was strong enough to preserve food instead of spoil it. One early method relates to a common experiment performed in schools where a base, usually sodium bicarbonate, is added to vinegar. The neutralization of the acid in the vinegar produces effervescence as carbon dioxide gas is liberated in the process. If the vinegar is more acid, more base is needed to fully neutralize it, and only then the effervescence stops. Lewis (1708–1781) introduced the first standardization of this process, which is known as acid–base titration [1]. This quantitative analysis consists in the addition of a base (or acid) solution of known concentration to the acidic (or basic) solution to be characterized. This standardization was possible thanks to papers impregnated in chemicals that change color with the acidity of the solution. Nevertheless, this process had to wait more than 150 years until better equipment was available to assess the acidity of a solution.

In 1936, Beckman and Fracker patented the first “Apparatus for testing acidity”, their description is the following [2]: “This invention relates broadly to electrical measuring instruments and has particular application in the measurement of potentials in circuits of extremely high resistance. A specific field in which it has great utility is in the determination of the hydrogen ion concentration (or pH as it is commonly termed) of solutions”. Proof that this invention was revolutionary is that Beckman is still a renowned brand for scientific equipment and pH meters are ubiquitous in almost all physics and chemistry laboratories.

The laboratory grade pH meters are usually quite reliable units that are typically under heavy use for decades. Usually only the standardized electrode probes have to be replaced when they are old or broken. It is the case of the old and reliable Metrohm 713 pH meter in our laboratory. One of the standard titration experiments that we perform, discussed in detail in section “Validation and characterization”, involves not only knowing the volume of base needed to neutralize a sample but also the rate at which it needs to be added to keep a pH constant. It is difficult to perform this kind of kinetic titration manually as the user needs to add precise amounts of base solution with a micropipette while keeping a written track of pH values and volumes added over time, with several measurements per minute for hour-long processes. Since this is a task that can be easily automated, there has been interest in the matter as reflected in books and papers on the issue published since 1959 [3], [4]. Nowadays, with the ever increasing presence of open hardware platforms, there are recent publications on similar automation relying on Arduino [5] and Raspberry [6] platforms. Moreover, pH meter manufacturers offer devices that attach to their units and perform these tasks automatically. The corresponding device provided by Metrohm is called Metrohm Titrando, and the simplest second hand units are listed over more than 5000$ online. The Metrohm Titrando automatically pumps a base (acid) solution into an acidic (basic) sample solution to keep a given pH constant, and accounts for the volume of base (acid) needed to maintain the pH constant over time. In contrast to sophisticated one-platform automation, we take here advantage of the output already provided by the Metrohm pH meter for the pH reading, and we adapted a high quality Open Source Pump project (OSPump) based on Arduino [7] to perform automatically the kinetic autotitration experiments, with a total cost under 100$.

## 2 Hardware description

This is a project intended to extend the capabilities of obsolete lab equipment in terms of being decades old, but still providing high quality measurements. There are comparatively less expensive units, in the range of several thousand dollars, that achieve this kind of kinetic autotitration, but at the cost of using lower quality pH meters. Therefore, our modified OSPump project not only is 50 times cheaper than the original manufacturer second hand units, but also enables to adapt the operation to many other potential measurements out of the scope of the commercial unit. Moreover, since it is based on the OSPump project [7], it can be used as an automated syringe pump whenever it is not in use as an OSPump Titrator.

• It extends the capabilities of obsolete high quality pH meters to perform autotitration for 50 times less cost than second hand commercial units.
• It provides a spare automated syringe pump when not in use as an autotitration device.
• It can be programmed to extend its capabilities with respect to commercial units.

**Fig. 1:**
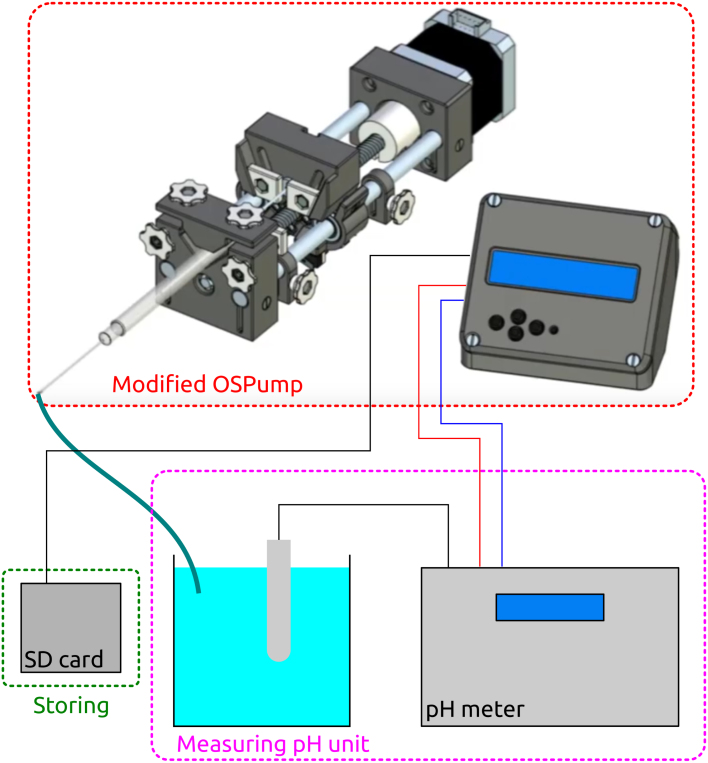
Scheme of the OSPump Titrator. The image of the OSPump was retrieved from the OSPump repository [7] under CC BY-SA 4.0 license: http://www.mass-spec.ru/projects/diy/syringe_pump/eng/.

As can be seen in Fig. 1, the device is composed of a modified OSPump [7], a SD card module to store the data, and the pH measuring unit. The main customizations that we performed to the original OSPump project were three: (i) remove the buzzer, (ii) use an analog reading pin in Arduino UNO to read the voltage from the pH meter analog output, and (iii) add the SD card module. Furthermore, the firmware was changed to operate as the OSPump Titrator, and it is provided in a Zenodo repository (see https://doi.org/10.5281/zenodo.7971517).

The analog output of the Metrohm 713 pH meter works in a particular way that conditions the setup that we need to perform in our device. This analog output was designed to be used as an output for a Metrohm 586 Labograph, a plotter that would draw on paper the pH values measured by the pH meter. This analog output can provide values ranging from −2 to 2 V with a resolution of 12 bits, while for practical purposes we do not deviate more than −1 to 1 V. Although the pH values can go easily from 1 to 12, the 12 bits resolution and range would produce inaccurate readings. Therefore, two parameters have to be set in the pH meter, and copied in the OSPump Titrator setup: (i) a pH at 0 V value that would define the pH at which the analog output gives a 0 V reading, and (ii) a pH 1 V range, which would mean the deviation from the pH that gives 0 V reading to provide a 1 V reading towards higher pH or −1 V towards lower pH. It is worth remarking that these voltages are outputs of the pH meter. Therefore, we will choose the 0 V reading usually 1 unit of pH below the desired pH to be maintained constant, and the 1 V range will be set to 1 unit of pH. Nevertheless, this means that if the measurement starts farther than 1 unit of pH from the pH set as 0 V value, it will become more and more unreliable as it departs from the 1 V pH range. We refer to the manual of the Metrohm 713 pH meter section “6.3. Applications of the analog output” available in the Metrohm website for further reading and schematics. Moreover, the analog input from the Arduino UNO used in this project only allows reading positive voltages, therefore a simple ＋1.65 V bias is applied by placing two 330 Ω resistors in series where one end is connected to the 3.3 V output from Arduino, the other end is connected to the analog output from the pH meter and the value sampled from the Arduino is the voltage between the two resistors. The Arduino UNO A/D converter is able to sample this voltage with 10 bits accuracy (a reading from 0 to 5 V with 1024 possible values). It might be possible to increase the resolution of the sensor reading by using a circuit based on an operational amplifier-based voltage level shifter with a gain of 1.25. In any case, for our purposes the resolution is good enough as shown in the video available in the supplementary repository (see https://doi.org/10.5281/zenodo.7971517), in which the readings from the pH meter and from the Arduino LCD panel can be compared in a real experiment. The differences are low and given the “noisy” nature of the measurement these differences are small enough.

Recording the data and storing it in a removable media as a microSD card is an important part of the OSPump Titrator, since it enables to know which it is the volume of solution injected over time to maintain a pH target value, and the actual pH being measured over time in an unattended way. *Firmware:*

*Available in the Zenodo repository*https://doi.org/10.5281/zenodo.7971517.

## 3 Design files summary

For the design files of the hardware parts of the OSPump we point the reader to the original repository of the Open Source Pump project http://www.mass-spec.ru/projects/diy/syringe_pump/eng/. For archiving purposes we have uploaded all necessary printing files and assembly instructions to the supplementary repository https://doi.org/10.5281/zenodo.7971517.

See Table 1

**Table 1:** Design files of the hardware parts with corresponding links to the Zenodo repository.

Design filename	File type	Open source license	Location of the file
Arduino_OSPump_Titrator_Operation_Validation	Video	CC-BY-SA	https://doi.org/10.5281/zenodo.7971517
SP_AssemblyInstructions	PDF file	CC-BY-SA	https://doi.org/10.5281/zenodo.7971517
Arduino_firmware_OSPump_Titrator	INO file	CC-BY-SA	https://doi.org/10.5281/zenodo.7971517
backSupport	STL file	CC-BY-SA	https://doi.org/10.5281/zenodo.7971517
carriage	STL file	CC-BY-SA	https://doi.org/10.5281/zenodo.7971517
caseBase	STL file	CC-BY-SA	https://doi.org/10.5281/zenodo.7971517
caseButtons	STL file	CC-BY-SA	https://doi.org/10.5281/zenodo.7971517
caseButtons_6buttons	STL file	CC-BY-SA	https://doi.org/10.5281/zenodo.7971517
caseover	STL file	CC-BY-SA	https://doi.org/10.5281/zenodo.7971517
caseover_6buttons	STL file	CC-BY-SA	https://doi.org/10.5281/zenodo.7971517
coupling	STL file	CC-BY-SA	https://doi.org/10.5281/zenodo.7971517
frontSupport	STL file	CC-BY-SA	https://doi.org/10.5281/zenodo.7971517
handKnob7mm	STL file	CC-BY-SA	https://doi.org/10.5281/zenodo.7971517
handKnob8mm	STL file	CC-BY-SA	https://doi.org/10.5281/zenodo.7971517
handKnob9mm	STL file	CC-BY-SA	https://doi.org/10.5281/zenodo.7971517
limitStop	STL file	CC-BY-SA	https://doi.org/10.5281/zenodo.7971517
plungerHolders	STL file	CC-BY-SA	https://doi.org/10.5281/zenodo.7971517
sideSyringeHolder	STL file	CC-BY-SA	https://doi.org/10.5281/zenodo.7971517
slider	STL file	CC-BY-SA	https://doi.org/10.5281/zenodo.7971517
topSyringeHolder12mm	STL file	CC-BY-SA	https://doi.org/10.5281/zenodo.7971517
topSyringeHolder25mm	STL file	CC-BY-SA	https://doi.org/10.5281/zenodo.7971517
wireHolder	STL file	CC-BY-SA	https://doi.org/10.5281/zenodo.7971517

**Bill of materials**

## 4 Bill of materials summary

**Fig. 2:**
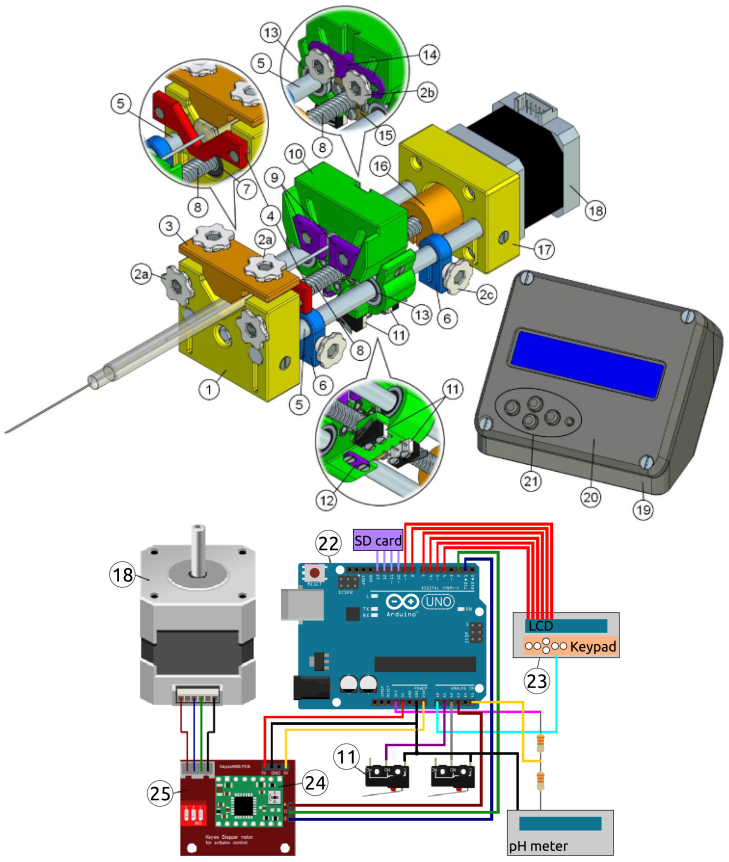
Schemes of the OSPump with part numbers corresponding to the ones in Table 2. Images adapted from the OSPump repository under CC BY-SA 4.0 license: http://www.mass-spec.ru/projects/diy/syringe_pump/eng/.

The materials in Table 2 from Part #1 to #26 are the same as the ones reported in [7]. We keep the numeration and names to be consistent with that publication, and have adapted the costs of all parts. Part #26, the active buzzer, was discarded in the OSPump Titrator, but it is included to be able to assemble the regular OSPump. Moreover, Parts #27, #28, and #29 are the cables and connectors to interface the pH meter, the SD card module, and the power supply for the OSPump Titrator, respectively. Fig. 2 shows schemes with the parts involved in this project. We do not include the plastic syringe, needle and plastic tubing as they are consumables available in the laboratory.

**Table 2:** List of components, cost, source, and material type.

Designator	Component	Number	Cost per unit	Total cost	Source of materials	Material type
Part #1	Front support^a^	1	1.1 $	1.1 $	3D printed	Plastic
Part #2	Hand knob^a^	8	0.03 $	0.24 $	3D printed	Plastic
Part #3	Top syringe holder^a^	1	0.3 $	0.3 $	3D printed	Plastic
Part #4	Side syringe holder^a^	1	0.2 $	0.2 $	3D printed	Plastic
Part #5	Guide rod (D = 8 mm)	2	3.95 $	7.90 $	3Dfilamento	Metal
Part #6	Limit stop^a^	2	0.5 $	1 $	3D printed	Plastic
Part #7	Bearing (688zz)	1	0.79 $	0.79 $	Solectro	Metal
Part #8	Lead Screw T8 50 cm L8	1	12 $	12 $	Solectro	Metal
Part #9	Plunger holders^a^	1	0.08 $	0.08 $	3D printed	Plastic
Part #10	Carriage^a^	1	1.2 $	1.2 $	3D printed	Plastic
Part #11	Endstop switch	2	1.49 $	2.98 $	Solectro	Composite
Part #12	Wire holder^a^	1	0.02 $	0.04 $	3D printed	Plastic
Part #13	Linear bearing (LM8SUU)	2	1.99 $	3.98 $	Solectro	Metal
Part #14	Slider^a^	1	0.05 $	0.05 $	3D printed	Plastic
Part #15	Trapezoidal Nut T8	1	3 $	3 $	Solectro	Metal
Part #16	Coupling^a^	1	0.3 $	0.3 $	3D printed	Plastic
Part #17	Back support^a^	1	1.3 $	1.3 $	3D printed	Plastic
Part #18	Stepper motor Nema 17	1	12.51 $	12.51 $	Solectro	Composite
Part #19	Case (base)^a^	1	2 $	2 $	3D printed	Plastic
Part #20	Case (cover)^a^	1	0.7 $	0.7 $	3D printed	Plastic
Part #21	Buttons^a^	5	0.05 $	0.05 $	3D printed	Plastic
Part #22	Arduino UNO	1	23.99 $	23.99 $	Solectro	Composite
Part #23	LCD Keypad shield	1	3.87 $	3.87 $	Solectro	Composite
Part #24	Stepper motor driver A4988	1	2.25 $	2.25 $	Solectro	Composite
Part #25	Control shield for A4988	1	1.82 $	1.82 $	Tecnoteca	Composite
Part #26	Active buzzer	1	0.59 $	0.59 $	Solectro	Composite
Part #27	Cables for the pH meter	1	2.99 $	2.99 $	Solectro	Composite
Part #28	SD card module	1	2.28 $	2.28 $	Solectro	Plastic
Part #29	Power supply 12 V	1	3.78 $	3.78 $	Solectro	Plastic

a Parts were designed by [7] and 3D printed on PLA, and estimated at 42 $/kg.

## 5 Build instructions

**Fig. 3:**
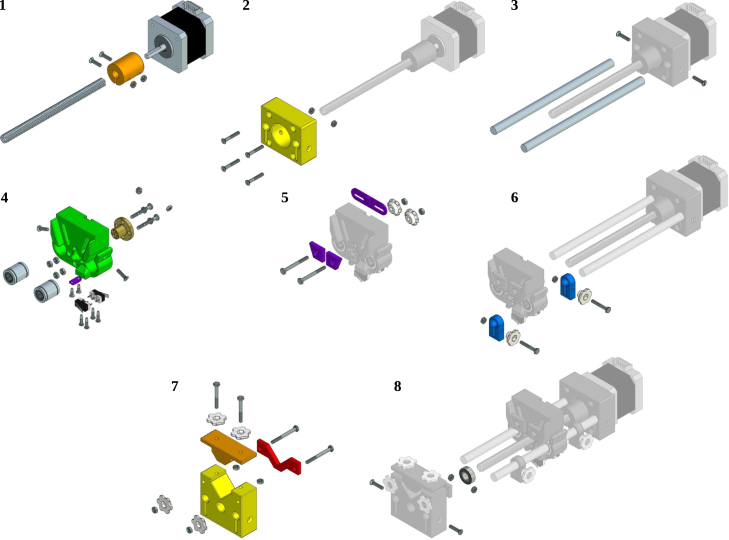
Steps to assemble the main body of the OSPump Titrator.

Adapted from http://www.mass-spec.ru/projects/diy/syringe_pump/eng/.

**Fig. 4:**
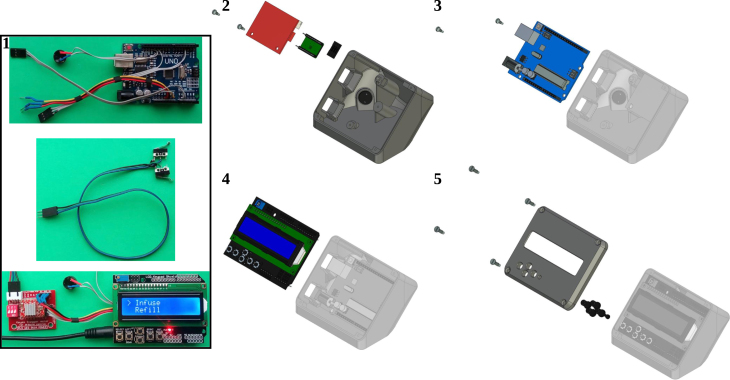
Steps to assemble the electronics and the LCD control panel of the OSPump Titrator.

Adapted from http://www.mass-spec.ru/projects/diy/syringe_pump/eng/.

For the instructions to build the OSPump the reader should refer to the original repository http://www.mass-spec.ru/projects/diy/syringe_pump/eng/, or the copy with detailed instructions in our repository in pdf format https://doi.org/10.5281/zenodo.7971517, since we followed all the available instructions detailed therein to build it. Therefore, here we will briefly describe the build instructions in Fig. 3, Fig. 4, for the main body and the LCD control display, respectively.

**Fig. 5:**
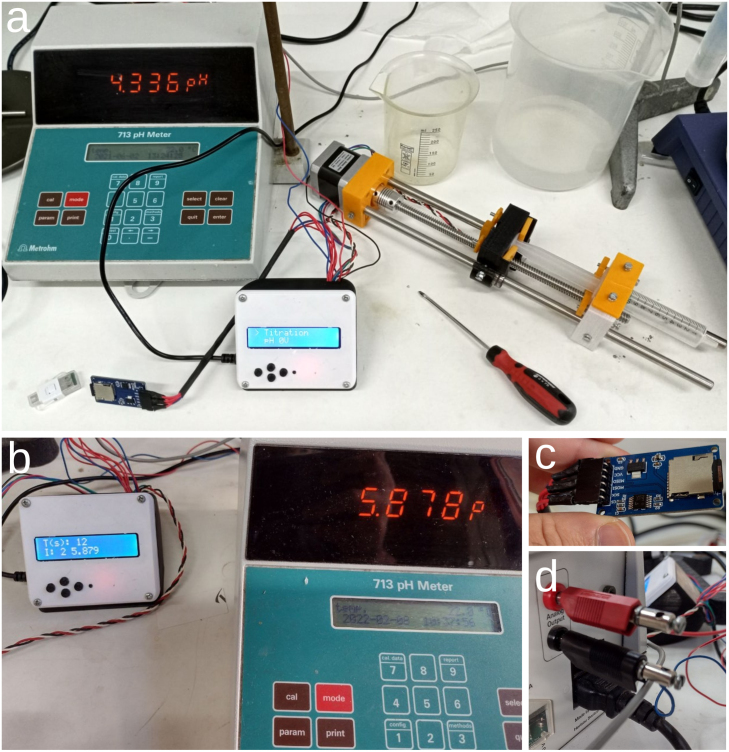
(a) OSPump Titrator based on Arduino [7]. (b) Detail showing a titration where the reading in the left LCD panel corresponds to the measurement from the Metrohm 713 pH meter on the right. (c) MicroSD card module to store the data of the quantity of NaOH solution pumped to keep a constant pH in the sample, keeping track of the pH and added volume over time. (d) Analog output from the pH meter used as input for the OSPump Titrator.

We describe now the adaptations implemented once the OSPump is ready and working. In Fig. 5a we show the final aspect of the OSPump Titrator, where the pH is monitored in both the pH meter and OSPump Titrator LCD panels as it can be seen in 5b. The first modification to the regular OSPump is to install the cables of the microSD card module, which will be accessible from the outside as can be seen in Fig. 5c. The second modification is to install the two cables that will go from the OSPump Titrator into the analog output of the pH meter, see Fig. 5d. The last steps corresponding to installing the syringe, needle, tubing and filling with the base titration solution, see Fig. 5a, are also common to the steps described in the OSPump manual [7].

## 6 Operation instructions

**Fig. 6:**
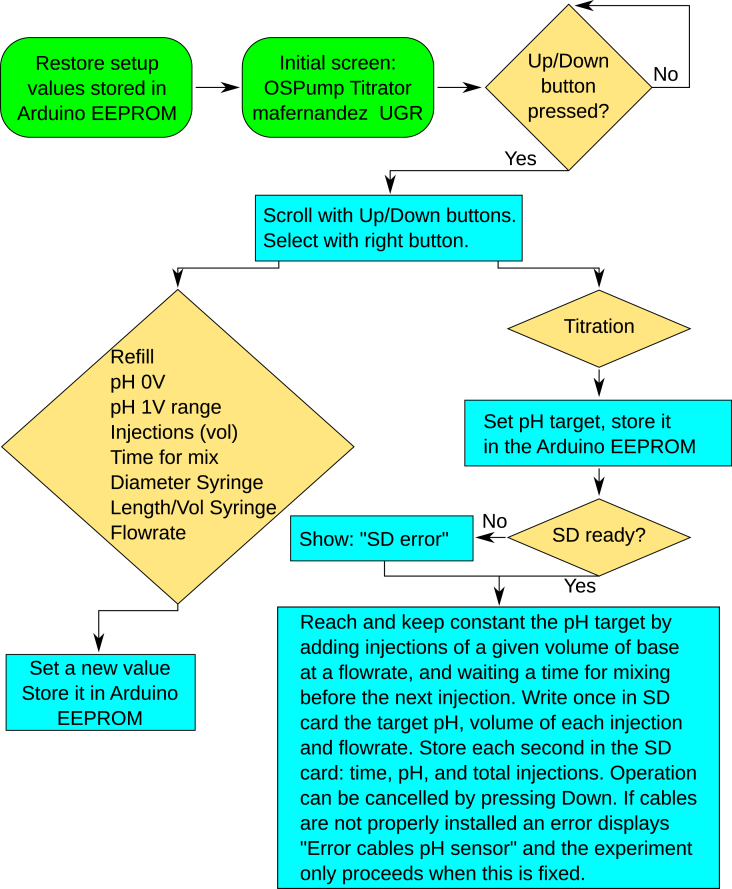
Flowchart of the operations that can be performed with the OSPump Titrator.

Before connecting the OSPump Titrator to the power outlet, the user needs to install the microSD card into the OSPump Titrator, and a syringe already loaded with a fresh base solution of known concentration, as shown in Fig. 5c and 5a, respectively. When the device is not powered, it is possible to slide and rotate by hand the lead screw to accommodate the syringe in it. In any case, it is possible to install an empty syringe since there is the option of refilling once it is powered as shown in Fig. 6. Once the syringe with the needle and tubing is placed in such a way that the tube is immersed in the sample and the OSPump Titrator is connected to the analog output of the Metrohm 713 pH meter (see Fig. 5d), the user needs to setup for the first time the pH 0 V and pH 1 V range both within the pH meter and the OSPump Titrator respective setup menus to make sure that both match, as explained in the Hardware description section. These values will be stored for future uses in both the pH meter and OSPump Titrator respective EEPROMs. Next, the user needs to setup either the diameter of the syringe or its Length-to-Volume ratio, since they are dependent on each other. Since the base will be added in discrete injections (aliquots), it is also possible to setup the volume of each aliquot, the flowrate and the time between dosing the aliquots. The way in which the values are introduced is inherited from the original OSPump firmware [7], where the value can be edited by pressing down. Then, each digit can be selected pressing left or right and the selected digit is incremented by pressing up or decreased by pressing down. When the user finishes, they have to press right until the value goes into non-editable mode and the value is stored in Arduino UNO’s EEPROM. Finally, the user will go into the Titration setup to specify the target pH. After checking if the microSD card and the cables to the pH sensor are ready and informing the user if they are not, it will start the titration according to the setup. This process will proceed until the user presses down to abort it or until one of the end-stops in the OSPump is pressed, in this case because the syringe would be empty. Furthermore, if the cables or connections to the pH meter are faulty during the measurement an error will be displayed and the measurement will only proceed once they are properly fixed. Once the user aborts the titration, they can press the left button in the OSPump Titrator to go back to the main menu. While we have added a functionality to constantly flush the text into the microSD card, ensuring that the data is written even if there is a power supply outage, it is advised that the user waits until the process is finished to remove the microSD card, to avoid any file corruption. Each new titration experiment is appended to the text file in the microSD card with the annotation “New Titration”. For this reason it is highly recommended to provide proper training and a comprehensive instructions manual along with this tool.

## 7 Validation and characterization

**Fig. 7:**
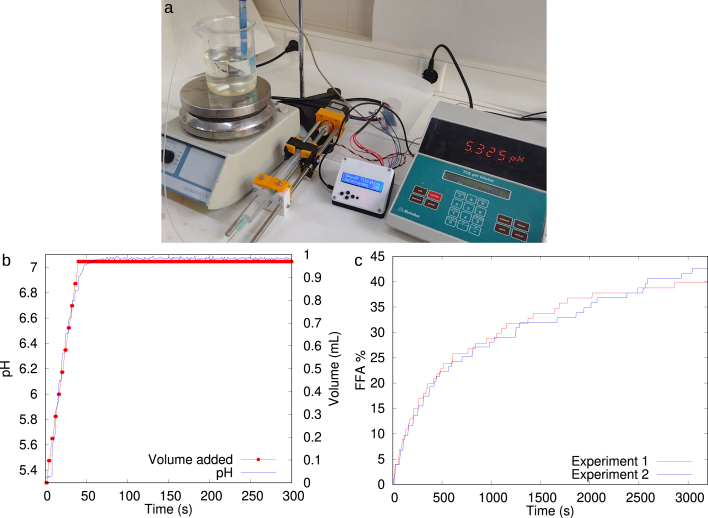
**(a)** Experimental setup ready to operate showing the pH probe on the beaker with the solution and a magnetic stirrer. **(b)** Automatic dosing of 0.1 N NaOH to reach pH 7 in a $1.13\cdot 10^{-3}$ M phosphate buffer. The same experiment was performed by hand by dosing 1 mL at once with a pipette, obtaining the same pH 7. Each dot corresponds to a pH check, and dosing if needed. Video of the experiment available in the supplementary repository (see https://doi.org/10.5281/zenodo.7971517). **(c)** Kinetic of release of FFA (%) of an olive oil in water emulsion stabilized by WPI measured with the OSPump Titrator. Two repetitions of the same experiment are shown in different colors. (For interpretation of the references to color in this figure legend, the reader is referred to the web version of this article.)

For the validation and characterization of the original OSPump as an automated syringe pump we refer to [7]. The OSPump Titrator is designed to maintain the pH constant in a sample solution by adding aliquots of sodium hydroxide (NaOH) in case of a reaction producing acidic conditions. The volume of each aliquot is selected by the user at the beginning of the experiment (see section Operating Instructions) and its concentration is known. In order to validate the autotitrator and demonstrate its accuracy, we designed an experiment where we knew in advance that a total of 1 mL of 0.1 N NaOH had to be dosed to a solution to obtain a pH 7. We compared doing it by hand and using our device. For this, we prepared a $1.13\cdot 10^{-3}$ M phosphate buffer. First, $38.98\cdot 10^{-3}$ g of NaH${}_{2}$PO${}_{4}\cdot$H${}_{2}$O (Scharlau) were dissolved in 0.25 L of MilliQ water. Next, the pH was adjusted manually adding 1 mL of a 0.1 N NaOH solution obtaining a pH 7. Then, the same measurement was performed by our device, as shown in Fig. 7a–b, starting at a low pH value and automatically dosing close to 1 mL of the 0.1 N NaOH solution, automatically stopping when the pH 7 was reached. In this case, 0.97 mL were added, well within the error standards of performing this procedure by hand. This experiment was recorded on video showing the operation of the device (see https://doi.org/10.5281/zenodo.7971517). We also demonstrate a more useful experiment in which we quantified the percentage of free fatty acids (FFAs) released into the aqueous solution when the lipase enzyme hydrolyzed triglycerides present in a vegetable oil in water emulsion, olive oil in our case, as shown in Fig. 7c. Olive oil is composed on between 99%–97% of triglycerides and the lipase activity consists of hydrolyzing triglycerides in a molecule of glycerol and one or two FFAs which decrease the pH of the aqueous solution. Both, olive oil and lipase are the protagonists in this method. First, an oil in water emulsion was prepared as a substrate for the lipolysis reaction, consisting of small droplets of oil dispersed in an aqueous solution containing an emulsifier (protein, surfactant, polymer, etc.). These emulsifiers adsorb onto the oil–water interface protecting oil droplets and stabilizing the emulsion by electrostatic and steric repulsion. In this case study, the protein used was Whey Protein Isolate (WPI), which presents negative charge at pH 7 and hence, stabilized the emulsion by electrostatic repulsion between oil droplets [8]. Next, the emulsion was used to measure the lipolysis, where the lipase is able to hydrolyze triglycerides from the olive oil, in one or two FFAs, decreasing the pH of the solution. The OSPump Titrator added NaOH aliquots to the solution where the reaction occurred. The activity of the lipase was directly proportional to the presence of FFAs in the solution. Hence, it was proportional to the amount of added NaOH. To measure this lipolysis, 4 ml of emulsion were needed. These were mixed with simulated intestinal fluid (SIF), adapted from the INFOGEST standard protocol (150 mM NaCl, 3 mM CaCl${}_{2}$, 1.13 mM NaH${}_{2}$PO${}_{4}$ at pH 7), and bile salts (5 mg/ml) [9]. At this point, the FFAs from the olive oil should be neutralized before starting the measurement of the lipolysis reaction. This was done by the OSPump Titrator, which maintains the pH at 7 during 30 min. Once the FFAs from the oil were neutralized, the operator added the lipase enzyme, in the form of pancreatin (1.6 mg/ml) or pure enzyme. Pancreatin is a mixture containing lipase and colipase enzymes, employed in the simulation of lipolysis *in vitro* as detailed in the standardized method by INFOGEST [9]. The OSPump Titrator maintained the pH at 7 by adding the required amount of 0.1 N NaOH, as the lipolysis proceeded in the system. A gentle magnetic stirring (300 rpm) was maintained during the experiment. This kind of titration experiment usually takes between 30 to 120 min [10], [11], [12], [13], and the endpoint can be found only after processing the data saved on the microSD card, as reflected by the pH being constant without addition of further NaOH aliquots. Finally, the % of released FFAs could be calculated by Eq. (1), where $V_{\mathrm{NaOH}}$ is the total added volume of NaOH, $N_{\mathrm{NaOH}}$ is the normality of NaOH used (0.1 N), $M_{lipid}$ is the average molecular weight of olive oil, $W_{lipid}$ is the total weight of olive oil used in the lipolysis reaction (calculated from 4 ml of emulsion), 2 means that one or two FFAs can be released from one triglyceride [14].

(1) $$\text{\%}FFA=100\frac{V_{\mathrm{NaOH}}\times N_{\mathrm{NaOH}}\times M_{lipid}}{W_{lipid}\times 2}$$

The OSPump Titrator is a valuable tool to measure the *in vitro* lipolysis of emulsified systems, where this process has to be performed twice. On one hand, prior to measuring the lipolysis, it is used to neutralize the FFAs present in the original olive oil. To this end, the OSPump Titrator is programmed to add NaOH aliquots (10–20 μl) until the target pH is maintained stable, which we consider happens when the operator sees that the pH is stable over 10 min. This process may last for 20–30 min. On the other hand, the lipolytic activity is measured by re-programming the addition of larger NaOH aliquots respect to the previous step. In Fig. 7c, an aliquot volume of 96.85 μl was selected at a flowrate of 50 μl/s. The OSPump Titrator automatically controlled the addition of NaOH to the lipolysis reaction, according to setup parameters. The pH meter monitored the pH, when this pH went below 0.1 of the target value, the OSPump Titrator added a new NaOH aliquot into the sample solution. The data was stored in the microSD card and could be analyzed by the operator after each measurement. In particular, the selected NaOH aliquot volume in each experiment was also stored in the microSD card, as it is needed to calculate the lipolysis activity. The number of aliquot injections and the record of pH values were also stored every second. Accordingly, the operator could easily calculate the volume of NaOH added over time. This allows to obtain the lipase activity using Eq. (1). Fig. 7c shows the obtained kinetics of release of FFAs from the *in vitro* lipolysis of olive oil in water emulsion stabilized by WPI in simulated intestinal fluid. These results obtained with the OSPump Titrator are in concordance with previous similar experiments performed manually [14].

This type of experiment provides insight into the ability of lipase to hydrolyze emulsified fat. The rate and extent of lipid hydrolysis in emulsified systems will depend on the properties of the emulsion. Hence, the type of emulsifier coating lipid droplets, the droplet size and susceptibility to coalescence and aggregation are all related variables influencing the rate and degree of lipolysis. The design of foods to control the bio-availability of lipids relies in monitoring the extent and rate of FFAs release as easily measured with the OSPump Titrator.

We can summarize the following main capabilities and limitations of our device, many of them directly inherited from the OSPump project [7]:

• Universal clamps for syringes with diameter up to 25 mm.
• All parameters can be changed using a keypad and are stored in the Arduino non-volatile memory: inner diameter of the syringe, flow rate, injections volume, target pH, pH 0 V and pH 1 V range, and time for mixing.
• Non-possible values due to discrete rotation of the stepper motor entered by the user are rounded to the nearest allowed value and displayed accordingly.
• Dispensing accuracy and reproducibility comparable to commercial syringe pumps.
• Stand-alone function storing the data in a microSD card.
• Automatic checking if the microSD card is properly installed and constantly flushing text to minimize lost data in any event.
• Automatic and constant checking if the cables to the pH meter are properly installed and holding the measurement until they are.
• The main limitation is the accuracy of the pH sensor reading. While we have enough accuracy for our measurements, the hardware can be upgraded to increase this accuracy.

## CRediT authorship contribution statement

**Teresa del Castillo-Santaella:** Investigation, Data curation, Visualization, Writing – original draft, Writing – review & editing. **Julia Maldonado-Valderrama:** Conceptualization, Writing – review & editing. **Miguel Angel Fernandez-Rodriguez:** Conceptualization, Methodology, Software, Validation, Writing – original draft, Writing – review & editing, Supervision, Funding acquisition.

## Declaration of competing interest

The authors declare that they have no known competing financial interests or personal relationships that could have appeared to influence the work reported in this paper.
